# Supplementary material for: Finding an Appropriate Mouse Model to Study the Impact of a Treatment for Friedreich Ataxia on the Behavioral Phenotype
Source: Genes (Basel). 2023 Aug 19;14(8):1654. doi: 10.3390/genes14081654 (PMC10454134; doi:10.3390/genes14081654)
Supplement: Supplementary file 1 [file genes-14-01654-s001.zip › genes-2539155-supplementary.pdf]

Supplementary Table 1: Sequences of primers.

PCR and qPCR primers

	Target	Name	F	R
Constuction plasmid	Intron px330	Hyb-In	TGCCTGAGAGCGCAGTCGAGAAGggagtcgctgcga cgctgc	TCCCCAACGTCCACATGGTGGCGGCTAGCCaa cctgaaaaaaagtgatttcag
		U6-shRE	GATCACGAGACTAGCCTCGAGCGGCCGCCCCCTTCACCGAGGGCCTATTTCCCATGATTCCTTCATATT TGCATATACGATACAAGGCTGTTAGAGAGATAATTGGAATTAATTTGACTGTAAACACAAAGATATTAGT ACAAAATACGTGACGTAGAAAGTAATAATTTCTTGGGTAGTTTGCAGTTTTAAAATTATGTTTTAAAATG GACTATCATATGCTTACCGTAACTTGAAAGTATTTCGATTTCTTGGCTTTATATATCTTGTGGAAAGGACG AAACACCGGACCGGTGCAGATTTGCTAGCAATTCTCGACCTCGAGACAAATGGCAGTATTCATCCACG CTACTTATCTACGTAGGGTGCAGCGGCCT	
		shRNA1	CCGGGCTGGACTCTTTAGCAGAGTTCTCGAGAAC TCTGCTAAAGAGTCCAGCTTTTTT	ctagaAAAAAGCTGGACTCTTTAGCAGAGTTC TCGAGAACTCTGCTAAAGAGTCCAGC
		shRNA3	ccggACGTGGCCTCAACCAGATTTGCTCGAGCAAA TCTGGTTGAGGCCACGTTTTTT	ctagAAAAAACGTGGCCTCAACCAGATTTGCT CGAGCAAATCTGGTTGAGGCCACGT
		shRNA4	ccggCCATACACGTTTGAGGACTATCTCGAGATAGT CCTCAAACGTGTATGGTTTTTT	ctagAAAAAACCATACACGTTTGAGGACTATCT CGAGATAGTCCTCAAACGTGTATGG
		shRNA6	ccgGTGGACCTAAGCGTTATGACTctcgagAGTCATA ACGCTTAGGTCCACtttttt	CTAGaaaaaaGTGGACCTAAGCGTTATGACTctc gagAGTCATAACGCTTAGGTCCA
		shRNA-scramble	ccggCCTAAGGTTAAGTCGCCCTCGctcgagCGAGG GCGACTTAACCTTAGGTTTTTT	ctagAAAAACCTAAGGTTAAGTCGCCCTCGCTC GAGCGAGGGCGACTTAACCTTAGG
PCR	mCherry		GGCCATCATCAAGGAGTTC	CCATGGTCTTCTTCTGCATTA
	AAV frataxine		GCTGTGGCAGGACTTCTGG	GCTTGTTCGGGGTTTGTTTA
qPCR	mCherry		TGAGGTCAAGACCACCTACA	GATGGTGTAGTCGTTGTG
qRT-PCR	Human frataxine		AAGCCATACACGTTTGAGGACTA	TTGGCGTCTGCTTGTTGATCA
	HPRT		CAGGACTGAAAGACTTGCTCGAGAT	CAGCAGGTCAGCAAAGAACTTATAGC

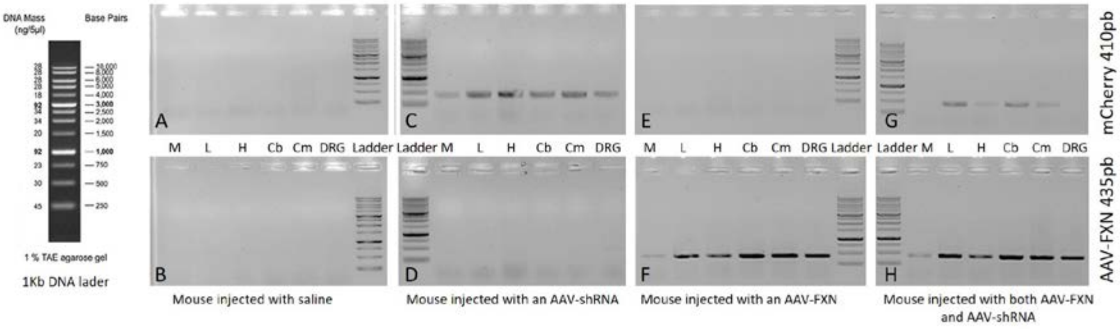

Supplementary Figure 1: For each mouse, two PCRs were made to confirm the treatment received by the presence of mCherry (shRNA) on the top and/or AAV-FXN on the bottom. Muscle (M), liver (L), heart (H), Cerebellum (Cb) and Cerebrum (Cm).

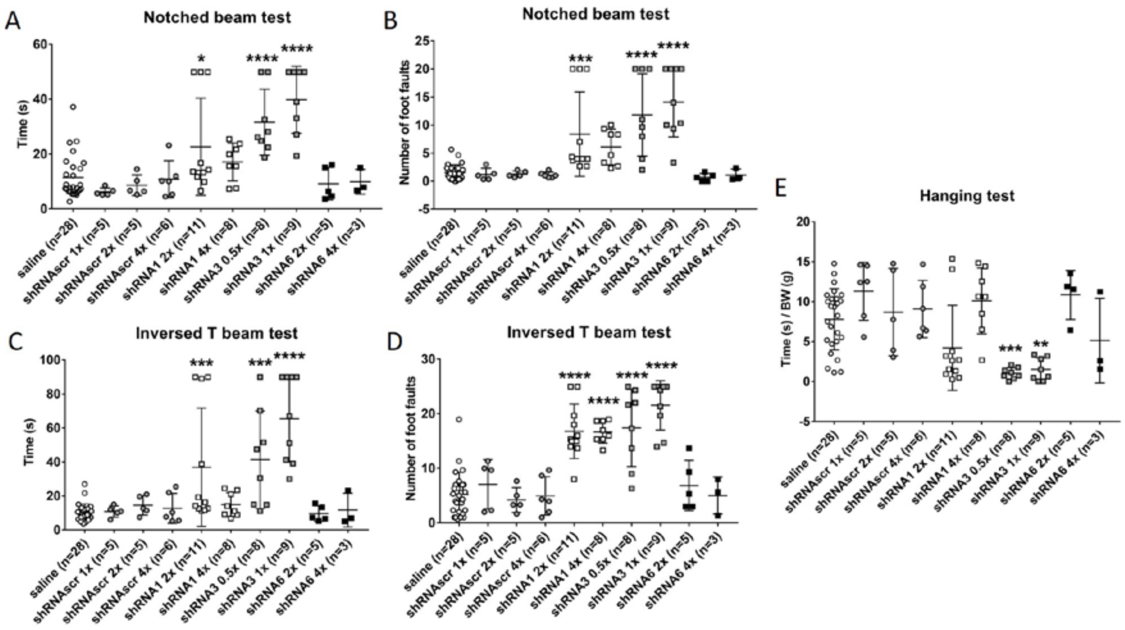

Supplementary Figure 2: The mice injected with an AAV containing different shRNAs were monitored with various behavior tests. The time taken by the YG8sR mice to cross the notched beam (**A**) and the inversed T beam (**C**) are illustrated 5 weeks after the injection. The number of foot faults made during the notched beam crossing (**B**) and the inversed T beam crossing (**D**) are also illustrated. In **E**, the hanging test is quantified as the ratio of hanging time/body weight. The results were analyzed by a two ways ANOVA test (Sidak's multiple comparisons test). **** p value < 0.0001 relative to the saline controls.
